# Quantitative Imaging Biomarkers of the Whole Liver Tumor Burden Improve Survival Prediction in Metastatic Pancreatic Cancer

**DOI:** 10.3390/cancers13225732

**Published:** 2021-11-16

**Authors:** Leonie Gebauer, Jan H. Moltz, Alexander Mühlberg, Julian W. Holch, Thomas Huber, Johanna Enke, Nils Jäger, Michael Haas, Stephan Kruger, Stefan Boeck, Michael Sühling, Alexander Katzmann, Horst Hahn, Wolfgang G. Kunz, Volker Heinemann, Dominik Nörenberg, Stefan Maurus

**Affiliations:** 1 Department of Medicine III, University Hospital, LMU Munich, Marchioninistr. 15, 81377 Munich, Germany; Julian.Holch@med.uni-muenchen.de (J.W.H.); Michael.Haas@med.uni-muenchen.de (M.H.); Stephan.Kruger@med.uni-muenchen.de (S.K.); Stefan.Boeck@med.uni-muenchen.de (S.B.); volker.heinemann@med.uni-muenchen.de (V.H.); 2 Fraunhofer Institute for Digital Medicine MEVIS, Max-von-Laue-Str. 2, 28359 Bremen, Germany; jan.moltz@mevis.fraunhofer.de (J.H.M.); horst.hahn@mevis.fraunhofer.de (H.H.); 3 CT R&D Image Analytics, Siemens Healthineers, Siemensstr. 1, 91301 Forchheim, Germany; alexander-muehlberg@hotmail.com (A.M.); michael.suehling@siemens-healthineers.com (M.S.); alexander.katzmann@siemens-healthineers.com (A.K.); 4 Department of Radiology, University Hospital, LMU Munich, Marchioninistr. 15, 81377 Munich, Germany; thomas.huber@umm.de (T.H.); Johanna.Enke@med.uni-muenchen.de (J.E.); Nils.Jaeger@med.uni-muenchen.de (N.J.); Wolfgang.Kunz@med.uni-muenchen.de (W.G.K.); Dominik.Noerenberg@medma.uni-heidelberg.de (D.N.); Stefan.Maurus@med.uni-muenchen.de (S.M.); 5 Department of Radiology and Nuclear Medicine, University Medical Center Mannheim, Theodor-Kutzer-Ufer 1-3, 68167 Mannheim, Germany

**Keywords:** computed tomography, radiomics, quantitative imaging biomarker, whole liver tumor burden, spatial analysis, metastatic pancreatic cancer, prognostic biomarker

## Abstract

**Simple Summary:**

Finding prognostic biomarkers and associated models with high accuracy in patients with pancreatic cancer remains a challenge. The aim of this study was to analyze whether the combination of quantitative imaging biomarkers based on geometric and radiomics analysis of whole liver tumor burden and established clinical parameters improves the prediction of survival in patients with metastatic pancreatic cancer. In this retrospective study a total of 75 patients with pancreatic cancer and liver metastases were analyzed. Segmentations of whole liver tumor burden from baseline contrast-enhanced CT images were used to derive different quantitative imaging biomarkers. For comparison, we chose two clinical prognostic models from the literature. We found that a combined clinical and imaging-based model has a significantly higher predictive performance to discriminate survival than the underlying clinical models alone (*p* < 0.003).

**Abstract:**

Finding prognostic biomarkers with high accuracy in patients with pancreatic cancer (PC) remains a challenging problem. To improve the prediction of survival and to investigate the relevance of quantitative imaging biomarkers (QIB) we combined QIB with established clinical parameters. In this retrospective study a total of 75 patients with metastatic PC and liver metastases were analyzed. Segmentations of whole liver tumor burden (WLTB) from baseline contrast-enhanced CT images were used to derive QIBs. The benefits of QIBs in multivariable Cox models were analyzed in comparison with two clinical prognostic models from the literature. To discriminate survival, the two clinical models had concordance indices of 0.61 and 0.62 in a statistical setting. Combined clinical and imaging-based models achieved concordance indices of 0.74 and 0.70 with WLTB volume, tumor burden score (TBS), and bilobar disease being the three WLTB parameters that were kept by backward elimination. These combined clinical and imaging-based models have significantly higher predictive performance in discriminating survival than the underlying clinical models alone (*p* < 0.003). Radiomics and geometric WLTB analysis of patients with metastatic PC with liver metastases enhances the modeling of survival compared with models based on clinical parameters alone.

## 1. Introduction

Pancreatic cancer (PC) is one of the major causes of cancer-related death and its high mortality rate has been unchanged for years [1]. Most patients are diagnosed with locally advanced or metastatic PC and the five-year survival rate is lower than 10% [2,3].

Finding prognostic biomarkers and associated models with high sensitivity and specificity for survival prediction remains a challenging problem [4]. Various studies have suggested prognostic biomarkers, such as levels of CA19-9, CRP and LDH, the ratio of neutrophils to lymphocytes, and performance status [5,6,7,8,9,10,11]. Moreover, several studies have evaluated clinical models for survival prediction in PC [5,6,12,13,14]. Xue et al. [6] designed a prognostic index model, helping to divide patients with metastatic PC into two risk groups in terms of survival, based on three clinical parameters (ECOG score, CA 19-9 level, and CRP level). Haas et al. [5] showed in a multivariable analysis of pretreatment prognostic factors statistical significance for the endpoint overall survival for the values log [CA 19-9], Karnofsky Performance Status (KPS; 90–100% vs. 60–80%), log [bilirubin] and log [CRP].

Besides genetic and biologic biomarkers, recent work suggests the use of radiomics signature and geometric measures of computed tomography imaging as biomarkers for the prediction of survival and therapeutic response [15,16,17,18,19]. Radiomics is an emerging field that extracts high-dimensional features from imaging data and uses them in statistical or machine-learning models with the goal of improving prediction accuracy in diagnosis and therapy responses [20,21]. Research data from radiomics studies on detecting quantitative imaging biomarkers (QIBs) for survival prediction in PC patients is scarce.

In order to estimate the overall survival or therapy response for different tumor types, in recent years, advances in automatic segmentation have enabled giving increasing consideration to analyzing the whole liver tumor burden (WLTB), instead of individual liver metastases, to more accurately assess holistic tumor heterogeneity [22,23]. Moreover, for patients with metastatic colorectal cancer, the tumor-burden score (TBS), assessing liver tumor burden, has become a prognostic tool [24,25]. The TBS is defined as the Pythagorean addition of the lesion number and the diameter of the largest lesion. This measurement was able to better estimate the survival of patients with colorectal liver metastases than the number of lesions or the diameter of the largest lesion alone [24,25]. Recently, the geometric metastatic spread (GMS) of the WLTB, i.e., the maximum spatial distance between liver metastases, has been found to be a promising predictor for survival in colorectal cancer patients [26]. The relevance of TBS and GMS of WLTB has not yet been evaluated for metastatic PC patients. Since PC is a tumor that frequently affects the liver in the metastatic situation, the WLTB seemed to be a suitable value for analysis. However, since the predictive value of radiomics or geometric analyses of WLTB for PC patients is unknown, we have compared the predictive performance of established clinical and quantitative imaging biomarkers based on WLTB in PC patients using a statistical approach.

The aim of this study was to investigate the prediction of one-year survival (1-YS) in patients with metastatic pancreatic cancer with the use of a systematic comparative analysis of quantitative imaging biomarkers (QIB) based on the geometric and radiomic analysis of whole liver tumor burden (WLTB) in comparison with predictions based on the tumor-burden score (TBS), WLTB volume alone, and two clinical models 

## 2. Materials and Methods

### 2.1. Patient Cohort

**Figure 1:**
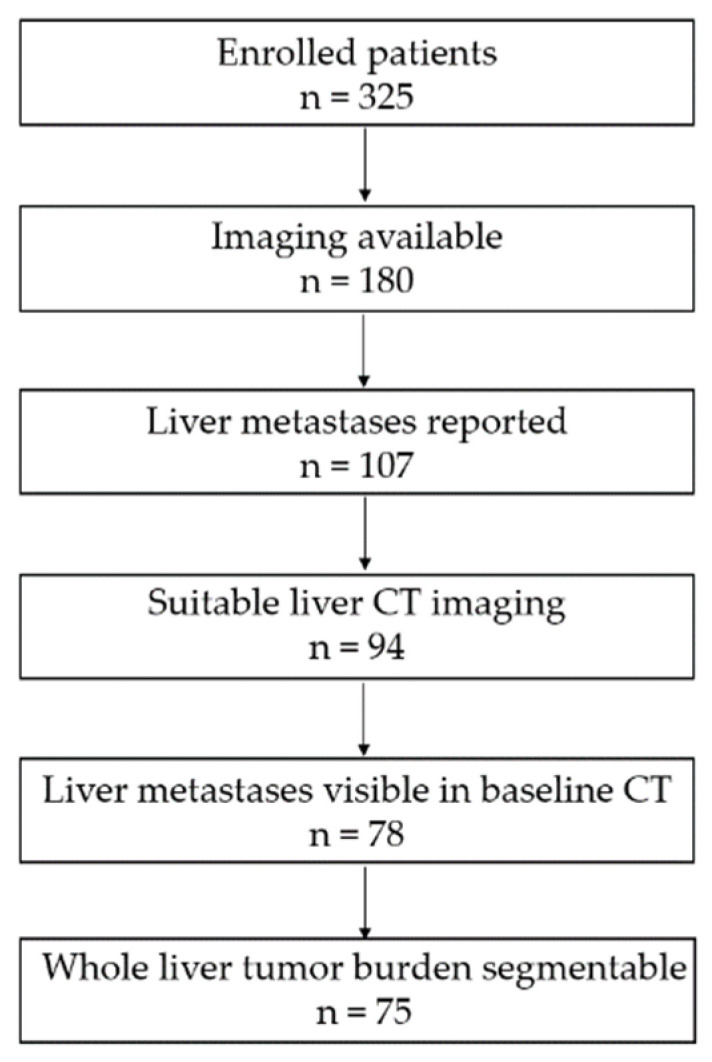
Flowchart illustrating patient exclusion criteria leading to the final study cohort of 75 metastatic PC patients.

The retrospective study was approved by and performed according to the guidelines of the local ethics committee (blinded). Written informed consent was obtained from all patients. We used a database of 325 patients with histologically proven metastatic PC from September 2015 to June 2017. For 180 patients, CT imaging data was available. Of these patients, 107 had reported metastatic liver disease. However, 13 patients had to be excluded because no contrast-enhanced CT imaging of the complete liver had been performed. In 16 further patients, no liver metastases were visible on baseline CT scans. Finally, three patients had to be excluded due to extensive metastatic liver disease that made a WLTB segmentation infeasible. The final study cohort consisted of 75 patients (Figure 1).

Clinical information (ECOG, CRP, Bilirubin, CA19-9) was obtained from the case files, information on survival data from the case file or register of residence. 

### 2.2. Imaging Studies

Baseline CT scans were acquired with various multidetector-row CT scanners from different manufacturers, using a default tube voltage of 120 kV. An intravenous contrast agent was applied in a weight-adapted manner. The images were acquired in portal venous phase and a standard soft-tissue kernel was used for the reconstruction. Slice thickness varied between 0.6 and 5 mm.

### 2.3. WLTB Segmentations and QIBs

Only contrast-enhanced CT scans were used for further evaluation. All CT scans were reviewed blinded to clinical data independently in a randomized manner. Screening for metastases was performed by two board-certified radiologists (blinded) with more than six years of experience in oncologic imaging. Automatic WLTB segmentations were created with a pre-trained U-Net [27] in a customized software based on MeVisLab (MeVis Medical Solutions, Bremen, Germany [28,29]). The U-Net was trained on images with a wide range of slice thicknesses and scanners and its performance does not depend on these factors. Both radiologists were able to revise the automatic WLTB segmentations, if necessary, by segmenting additional tumors or adapting tumor contours.

Imaging features were extracted using MeVisLab and PyRadiomics [30]. These features include previously discovered QIBs, such as the mean attenuation and the volume, both absolute and as a percentage of the liver volume, the TBS, and bilobar disease, i.e., presence of metastases in both the left and right functional liver lobes. Moreover, we evaluated the established four-feature radiomics signature described by Aerts et al. [21] for the WLTB because of the good predictive performance of this model when applied to target lesions in multiple oncological imaging studies [20,30,31,32]. These four quantitative image features (energy, compactness, GLRLM nonuniformity, wavelet nonuniformity) describe the tumor heterogeneity and compactness and have been defined by the Image Biomarker Standardization Initiative [33].

Additionally, we also evaluated the spatial geometric distribution of the tumors within the liver with geometric metastatic spread (GMS) features [26]: they measure the maximum distance of liver metastases along the three scanner axes (MSx, MSy, MSz) as well as the surface-area-to-volume ratio (SA/V), which quantifies the dispersion of metastases within the liver.

### 2.4. Clinical Baseline Models

Two models for survival prediction in PC, based on clinical parameters from the literature, were used as a baseline for investigating the benefit of adding WLTB QIBs. Both are multivariable Cox proportional hazard models and use those baseline parameters that were found to be significant in a multivariable Cox model in the original studies. The first model is based on results by Haas et al. [5] and uses

- ECOG [34] 0 vs. ECOG 1-2 at the time of diagnosis of metastatic PC; this is roughly equivalent to the Karnofsky performance score (KPS) 90–100% vs. 60–80%, as used in the original study [5]
- Baseline log(CRP), where the natural logarithm is applied to the continuous value of CRP in mg/dl
- Baseline log(bilirubin), analogously

The stage of disease (locally advanced vs. metastatic pancreatic cancer) is omitted from the original model because we consider metastatic patients only. A CPH model with these three parameters was fitted to our data and the concordance index (C-index) was computed to estimate the predictive performance.

The second model uses baseline parameters from the prognostic index model of Xue et al. [6]:

- ECOG 0-1 vs. two at the time of diagnosis of metastatic PC
- Baseline CA19-9 ≥1000 U/mL vs. <1000 U/mL
- Baseline CRP ≥5 mg/L vs. <5 mg/L
- Again, a CPH model is fitted and evaluated by the C-index.

### 2.5. Statistical Analysis

Survival time was defined as the time from the date of the first baseline CT at the initial diagnosis of metastatic disease to the date of death (if applicable). Descriptive statistics, such as median and range for continuous variables and frequencies for categoric variables, were used to summarize the data and features defined above.

**Figure 2:**
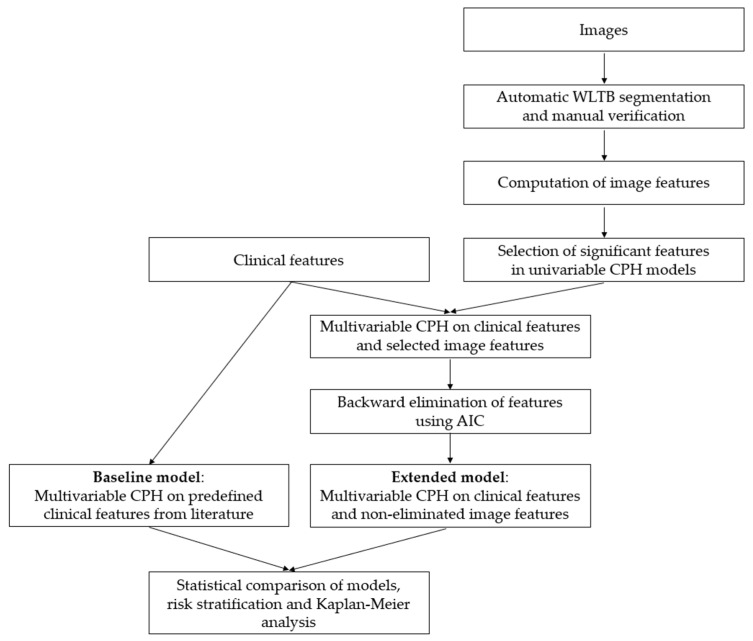
Flowchart illustrating the statistical analysis pipeline.

Univariable CPH models were computed for all image-based features. Significant features (*p* < 0.2, Wald test) were candidates for being combined with parameters from the clinical baseline models into a multivariable CPH model. Backward elimination, based on the Akaike information criterion (AIC) was used to select the final parameter set for the combined models. The likelihood ratio (LR) test was used to compare the C-indices of models. This test requires nested models, i.e., the parameters in one model must be a subset of the parameters of the other model. Therefore, all clinical parameters from the baseline model were kept in the combined model. For the final model, high and low risk groups were defined by splitting the cohort at the median survival. Survival differences between the two groups were visualized by a Kaplan–Meier curve and quantified by the log-rank test. The whole analysis pipeline is illustrated in Figure 2.

For a deeper understanding of the features, a univariable Spearman correlation heatmap and dendrogram were generated to quantify associations between clinical and imaging variables.

Data analysis was done with R (version 3.6, packages survival, glmnet, MASS) and Python (version 3.7.1, packages pandas, numpy, scipy, lifelines). Academic radiologists had full control over the data and no product or commercial software was tested in the current study.

## 3. Results

### 3.1. Demographic Data

Baseline characteristics of the included patients are shown in Table 1.

**Figure 3:**
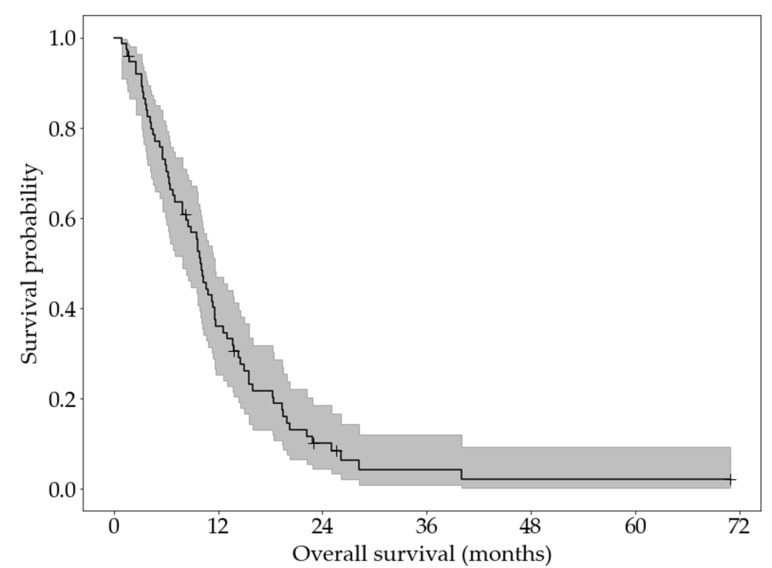
KaplanMeier curve and 95% confidence interval of the overall survival of patients of 75 included metastatic PC patients.

A Kaplan–Meier curve of overall survival is shown in Figure 3. The median survival was 10.0 months (95% confidence interval: 7.9 to 11.6 months). Six patients were right-censored.

### 3.2. WLTB Segmentations

**Figure 4:**
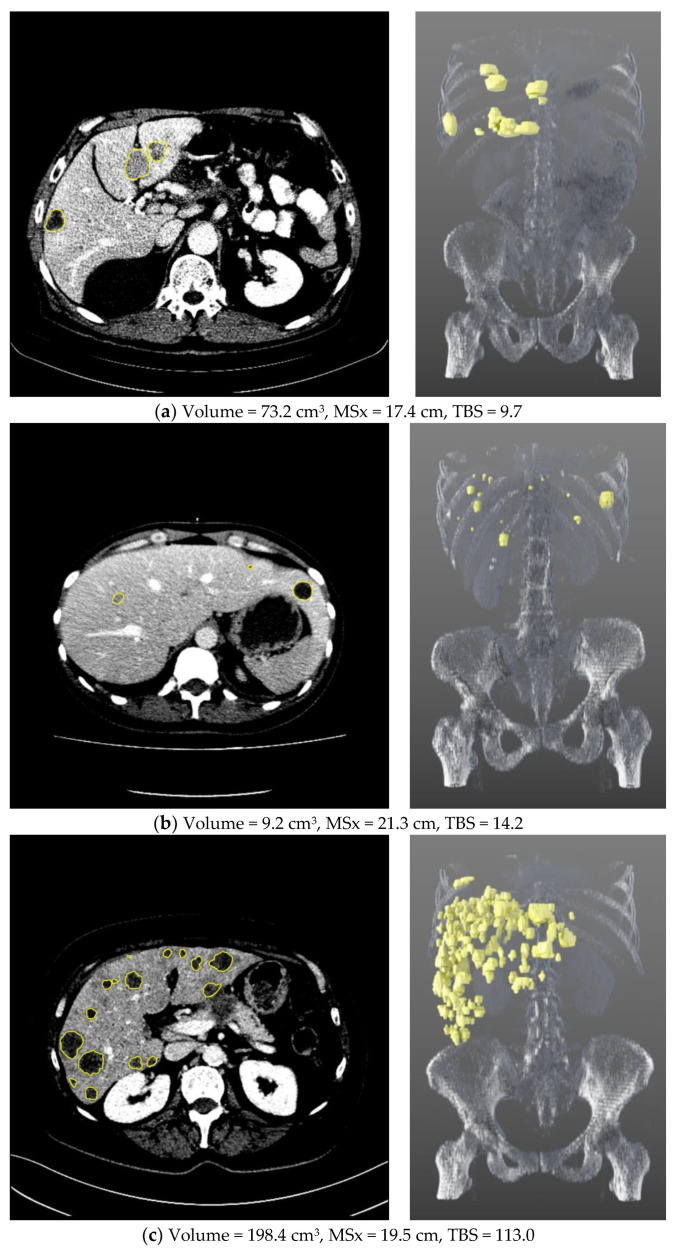
Three examples of metastatic PC patients with segmented WLTB, visualized in 2D (left) and 3D (right). These patients represent (**a**) longest (still alive after 70.9 months), (**b**) median (died after 9.9 months), and (**c**) shortest overall survival (died after 0.9 months).

In total, 1135 liver metastases were segmented, ranging from 1 to 113 (median 7) per patient. In Figure 4, three representative patients spanning the range of overall survival are shown. A comparison of Figure 4a,b illustrates the different WLTB parameters. While the patient who lived longest (a) has a larger WLTB volume, the median patient (b) has a higher number of metastases. Also, the GMS is different between them: patient (b) has a larger metastatic spread on the frontal axis (MSx), i.e., metastases are located over the whole width of the liver, whereas in patient (a) metastases are predominantly located within the right liver lobe, resulting in a lower MSx. Patient (b) also has a higher tumor burden score, which, in this case is dominated by the number of metastases, although patient (a) has larger metastases. The patient shown in Figure 4c exceeds the others in both WLTB volume and number of metastases, but still has a lower MSx than patient (b).

### 3.3. Survival Models

The univariable CPH models for WLTB parameters are shown in Table 2. Of all 13 imaging-based parameters, 8 were significantly associated with survival. 

**Figure 5:**
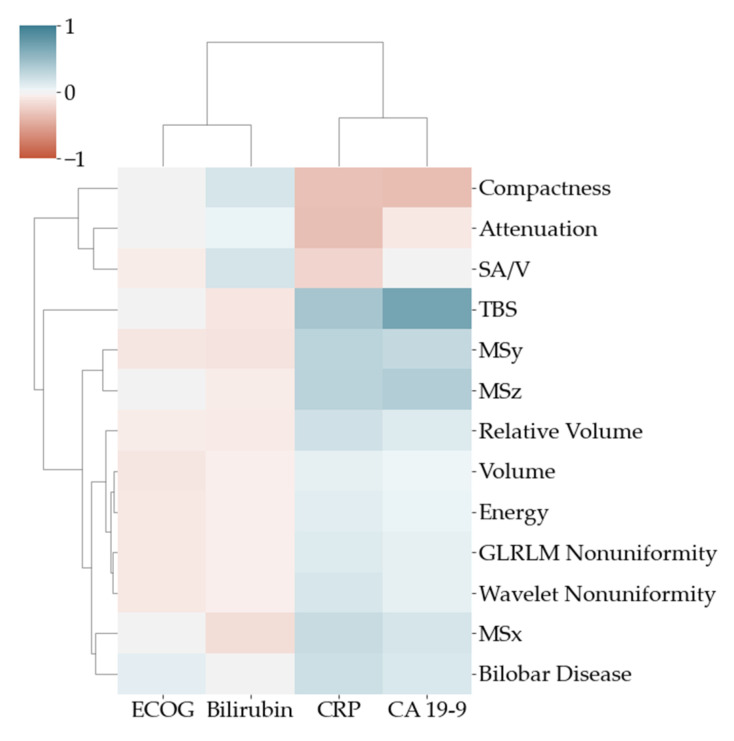
Pearson correlation heatmap with dendrogram to visualize the association between clinical (*x* axis) and imaging (*y* axis) variables.

To visualize the univariable associations of imaging and clinical variables, a correlation heatmap with dendrogram is shown in Figure 5. The highest association between clinical parameters and imaging was found between CA 19-9 and TBS. 

Table 3 summarizes the results of the clinical baseline models and the models extended with WLTB parameters.

For both baseline models, C-indices of roughly 0.62 were found. The only significant parameter was ECOG in the model of Xue et al. [6].

For both models, WLTB volume, TBS, and bilobar disease (plus energy in the Haas et al. model, respectively) were the remaining WLTB parameters after backward elimination and were thus used in the extended multivariable models (extended model 1 based on [5], extended model 2 based on [6]). This resulted in C-indices of 0.736 and 0.699, which are both significantly higher than those of the respective baseline models. TBS and bilobar disease were the only significant parameters in both final models.

**Figure 6:**
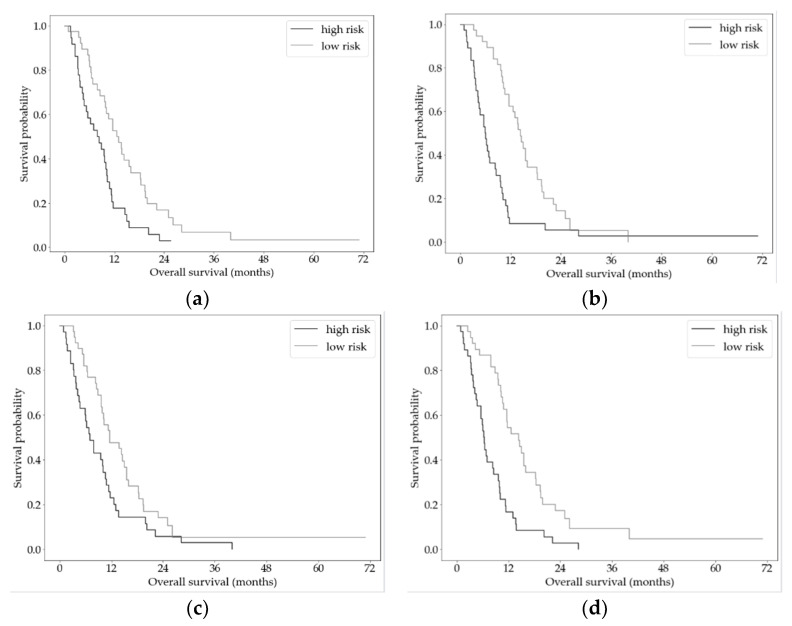
Kaplan–Meier curves for overall survival for high- and low-risk PC groups stratified by the baseline and extended CPH models. (**a**) Baseline model based on [5]. (**b**) Extended model 1 based on [5]. (**c**) Baseline model based on [6]. (**d**) Extended model 2, based on [6].

Both extended models (extended models 1 and 2) were used to stratify patients into high- and low-risk groups with significantly different survival (*p* < 0.0001). The Kaplan–Meier curves are shown in Figure 6. Median survival in the risk groups was for the extended model 1 (b) 6.0 vs. 14.3 months and for the extended model 2 (d) 6.4 vs. 14.3 months. For comparison, we also show the Kaplan–Meier curves for risk stratification using the baseline models.

## 4. Discussion

In this work, we investigated whether the whole liver tumor burden (WLTB) and especially different quantitative imaging biomarkers (QIB) of WLTB could be used as prognostic biomarkers in patients with metastatic pancreatic cancer (PC).

We therefore analyzed contrast-enhanced CT scans of 75 patients with PC and liver metastases scheduled for first-line therapy. In detail, we compared different WLTB-based imaging biomarkers including already discovered discriminative QIBs found in oncological imaging with two clinical baseline models [5,6] for predicting overall survival. Our goal was to explore if potential and robust predictors of patient survival could be identified based on the quantitative imaging in baseline CT scans, which may serve as early imaging biomarkers for risk stratification. In this study, for both clinical baseline models, C-indices of roughly 0.62 were found. After the addition of WLTB imaging parameters, the C-index increased from 0.62 to 0.74. These extended models (1 and 2) had a significantly higher predictive performance in discriminating survival than the underlying clinical models alone (*p* < 0.003). The results of this study suggest that WLTB imaging parameters are informative in terms of predicting survival in patients with metastatic PC.

Patients with PC have a very poor prognosis. The identification of specific tumor characteristics associated with prognosis is important for treatment decisions. Several studies with different cancer entities have already applied radiomics for survival prediction [18,19,20,21,32,36,37,38,39,40]. Until now, there are only a few other studies on the prognostic utility of imaging parameters in PC [15,16,17,18,19]. For example, in a study by Attiyeh et al. [17], an association between the quantitative analysis of 255 texture features from the primary tumor region representing different aspects of image heterogeneity and survival could be shown for patients with resected PC using two models with clinical data (CA 19-9, Brennan-Score) and radiomics features. Another study by Chakraborty et al. [18] showed an association between a model of 255 well-established first- and second-order intensity and edge-based features and the prediction of two-year survival for patients with resectable PC. Moreover, in a study by Parr et al. [19] a six-feature radiomics signature of primary pancreatic tumors was identified that achieved better overall survival prediction performance than the clinical model alone for patients undergoing stereotactic body radiotherapy. Since there are some studies on the prognostic utility of imaging parameters of primary pancreatic tumors, research data of radiomics studies to detect QIBs for survival prediction for patients with PC and liver metastases are warranted.

To the best of our knowledge, this work is the first study that compared WLTB, the percentage of total tumor in the liver, and especially WLTB-based imaging biomarkers for predicting survival in patients with metastatic PC. In this study, segmentations of WLTB from baseline contrast-enhanced CT images were used to derive different QIBs. The extracted WLTB imaging features include the whole liver tumor burden volume, bilobar disease, i.e., presence of metastases in both left and right functional liver lobes, four features of the established radiomics signature by Aerts et al. [21], and the geometric metastatic spread (GMS).

We decided to use WLTB because it has been shown to be an independent predictor of prognosis in patients with metastatic colorectal cancer and for other solid tumors [22,23]. Since PC is a tumor that frequently affects the liver in the metastatic situation, the WLTB seemed to be a suitable value for analysis. Since there are no established biomarkers of WLTB for patients with PC to date, we decided to use these WLTB QIBs, which are reliable predictive biomarkers and show higher predictive values than clinical parameters in patients with hepatic metastatic colorectal cancer in a study by Mühlberg et al. [26]. Since the added predictive value of radiomics or geometric analysis of WLTB for patients with metastatic PC is unknown, we compared the predictive performance of established clinical and quantitative imaging biomarkers based on WLTB, using a statistical approach. In the extended models, WLTB volume, TBS, bilobar disease and energy (in the extended model 1) were the WLTB parameters that were kept, by backward elimination, and were thus used in the extended multivariable model.

The TBS, incorporating maximum tumor size and number of lesions, was analyzed for survival discrimination in metastatic colorectal cancer patients and was outlined as an accurate tool to account for the impact of tumor morphology on long-term survival [24]. In a study by Mühlberg et al. [26] the TBS represents a reliable predictive QIB and shows higher predictive values than all clinical models for patients with metastatic colorectal cancer. Furthermore, TBS was strongly associated with the risk of recurrence after resection in patients with HCC and pancreatic neuroendocrine tumors [41,42,43].

We applied the TBS for our collective patients with PC and a significant correlation between TBS and prognosis was shown. Therefore, TBS seems to be a suitable prognostic marker for patients with metastatic PC. External validation cohorts and further studies are needed to clarify the prognostic role of TBS in patients with PC and liver metastases.

In addition to TBS, GMS (except SA/V) was significantly associated with survival in the univariable CPH models. However, TBS performed best in multivariable statistics and GMS contained no remaining WLTB parameters, after backward elimination, and was not used in the extended multivariable models. As in our study, TBS and GMS were features with significant discriminative performance in univariable statistics in the study by Mühlberg et al. [26]. Moreover, in that study TBS also showed the best discriminative performance whereas GMS was superior to TBS in the machine-learning approach [26].

Moreover, we evaluated the four features (energy, compactness, GLRLM nonuniformity, wavelet nonuniformity) of the established radiomics signature by Aerts et al. [21]. Aerts et al. [21] defined a four-feature signature by focusing on the most robust features for prognostication in a lung dataset, and validated their signature using independent lung, head or neck cancer patient cohorts. The performance of the four features appears plausible, since this data has often been shown to be of high and reproducible predictive value across various cancer types [21,25,26,27,31,32]. In this study, all parameters were significantly associated with survival in the univariable CPH models for WLTB parameters. Energy was, in the Haas et al. case, a remaining WLTB parameter after backward elimination and was used in the extended multivariable model. External validation cohorts and further studies are needed to find out the prognostic role of the established radiomics signature by Aerts et al. in PC.

Our study had some potential limitations. First, the study is only of medium sample size. Second, no external validation cohort was available. External validation cohorts and further prospective studies are needed to determine the prognostic role of WLTB-derived QIBs. Another problem may arise from the variety of different CT scan protocols, especially for texture quantifications. Another limitation of the segmentation of WLTB is that it can only be used if liver metastases can be detected with the currently available software. In this study, 16 patients had to be excluded because no liver metastases were visible in baseline CT imaging and three patients had to be excluded because they were so extensively metastasized that a WLTB segmentation was not feasible. According to the current state of technology, its use is therefore not possible in all patients. However, despite the variety of different CT-scan protocols in this study, in most of the cases, segmentation of the WLTB was possible. 

Combined clinical and imaging-based risk scores using radiomics may be useful in the future for patient risk stratification and the support of treatment decisions in patients with metastatic PC. Currently, WLTB is not analyzed in clinical routine, however, appropriate software is under development [27], so an application in clinical routine would be possible in the future. This makes the WLTB and WLTB QIBs an interesting tool to be used as prognostic biomarkers in patients with metastatic PC.

## 5. Conclusions

Clinical models for predicting survival in metastatic PC have limited prognostic value. WLTB–based measures from CT-images such as whole liver tumor volume, tumor burden score, bilobar disease and radiomics features, such as energy, are significantly associated with the outcome of patients with metastatic PC. Therefore, extending established clinical models with the image-based parameters of WLTB significantly increases prognostic value. The results of this study conclusively underline that the roles of WLTB and QIBs of the WLTB as potential predictive biomarkers for survival require further study.

## Figures and Tables

**Table 1:** Baseline characteristics of 75 included metastatic PC patients. ns: not specified. Continuous variables are given as median and range. T stage/N Stage according to AJCC (American Joint Commission on Cancer), tumor grading according to [35].

Characteristic	Value	
No. of patients	75	
age (y)	64 (41–83)	
sex	male	50 (66.7%)
	female	25 (33.3%)
primary tumor location	head	26 (34.7%)
	body	15 (20.0%)
	tail	13 (17.3%)
	ns	21 (28.0%)
tumor grading	G1	1 (1.3%)
	G2	12 (16.0%)
	G3	59 (78.7%)
	G4	3 (4.0%)
T stage	T1	1 (1.3%)
	T2	9 (12.0%)
	T3	46 (61.3%)
	T4	19 (25.3%)
N stage	N0	19 (25.3%)
	N1	55 (73.3%)
	N2	1 (1.3%)
CA 19-9 (U/mL)	463 (0–422,000)	
CEA (ng/mL)	6.3 (0.2–1697.0)	
CRP (mg/dL)	1.0 (0.0–18.2)	
LDH (U/L)	226 (128–937)	
bilirubin (mg/dL)	0.6 (0.3–39.8)	
ECOG	0	33 (44.0%)
	1	38 (50.7%)
	2	3 (4.0%)
	3	1 (1.3%)
overall survival (d)	304	
whole liver tumor burden volume (cm^3^)	9.3 (0.3–2832.5)	
affected liver lobes	left	61 (81.3%)
	right	67 (89.3%)
	both	53 (70.7%)

**Table 2:** Univariable CPH models for WLTB parameters with hazard ratio (HR), 95% confidence interval (CI), and *p*-value (Wald test). * Parameters with *p* < 0.2 were candidates for extended models.

Parameter	HR [CI]	*p*
attenuation (HU)	1.00 (0.98–1.01)	0.632
volume (cm^3^)	1.00 (1.00–1.00)	0.127 *
relative volume (%)	0.99 (0.96–1.02)	0.428
TBS	1.02 (1.01–1.03)	0.002 *
bilobar disease	2.25 (1.31–3.84)	0.002 *
energy	1.00 (1.00–1.00)	0.196 *
compactness	0.09 (0.02–0.44)	0.002 *
GLRLM nonuniformity	1.00 (1.00–1.00)	0.203
wavelet nonuniformity	1.00 (1.00–1.00)	0.283
MSx (cm)	1.03 (1.00–1.07)	0.083 *
MSy (cm)	1.04 (1.00–1.08)	0.081 *
MSz (cm)	1.05 (1.01–1.10)	0.018 *
SA/V (1/cm)	1.03 (0.92–1.14)	0.618

**Table 3:** Multivariable CPH models with hazard ratio (HR), 95% confidence interval (CI), *p*-value (Wald test) and C-index for clinical baseline models and models extended with WLTB parameters as well as *p*-value for C-index of extended model vs. baseline model (likelihood ratio test): * significant (*p* < 0.05).

Parameter	Baseline Model (Haas et al. [5])			Extended Model 1		
	HR (CI)	*p*	C-Index	HR (CI)	*p*	C-Index
ECOG 0 vs. 1–3	1.59 (0.98–2.57)	0.06	0.614	1.49 (0.91–2.42)	0.11	0.736 (*p* = 0.0003 *)
log(CRP)	1.00 (0.97–1.03)	0.99		0.99 (0.96–1.02)	0.35	
log(Bilirubin)	1.18 (0.98–2.57)	0.13		1.16 (0.93–1.45)	0.20	
volume [cm^3^]				1.00 (1.00–1.00)	0.06	
TBS				1.01 (1.00–1.03)	0.04 *	
bilobar disease				2.02 (1.09–3.75)	0.03 *	
energy				1.00 (1.00–1.00)	0.10	
**Parameter**	**Baseline Model (Xue et al. [6])**			**Extended Model 2**		
	**HR (CI)**	***p***	**C-Index**	**HR (CI)**	***p***	**C-Index**
ECOG 0–1 vs. 2	3.75 (1.27–11.14)	0.02 *	0.621	2.79 (0.94–8.30)	0.07	0.699 (*p* = 0.003 *)
CA19-9 ≥ 1000 [U/mL]	1.58 (0.98–2.55)	0.06		1.01 (0.57–1.77)	0.98	
CRP ≥ 5 [mg/dl]	1.53 (0.77–3.06)	0.23		1.52 (0.76–3.05)	0.24	
volume [cm^3^]				1.00 (1.00–1.00)	0.22	
TBS				1.02 (1.00–1.03)	0.03 *	
bilobar disease				1.86 (1.02–3.37)	0.04 *	

## Data Availability

The data presented in this study can be made available upon request from the corresponding author.
